# Transdiagnostic remission of psychiatric comorbidity in post-traumatic stress disorder, ADHD, and binge-eating disorder using ketogenic metabolic therapy: a retrospective case report

**DOI:** 10.3389/fnut.2025.1600123

**Published:** 2025-06-23

**Authors:** Erin L. Bellamy, Nicole Laurent

**Affiliations:** ^1^School of Psychology, University of East London, London, United Kingdom; ^2^Family Renewal, Inc., Vancouver, WA, United States

**Keywords:** ketogenic diet, ketogenic metabolic therapy, post-traumatic stress disorder, PTSD, case report, metabolic psychiatry, ADHD, binge-eating disorder

## Abstract

**Background:**

Psychiatric comorbidities, including post-traumatic stress disorder (PTSD), ADHD, and binge-eating disorder (BED), frequently share overlapping symptoms and metabolic dysfunctions. Disorder-specific treatments may not adequately address these shared biological mechanisms, resulting in suboptimal outcomes. This case report evaluates ketogenic metabolic therapy (KMT) as an intervention specifically targeting these transdiagnostic features.

**Methods:**

A 38 years-old female with PTSD, ADHD, BED, bipolar II disorder, depression, anxiety, and premenstrual dysphoric disorder diagnoses participated in a structured 8 weeks KMT psychoeducation program, with ongoing weekly professional and peer support up to 24 weeks. Standardized assessments, including the PHQ-9, GAD-7, DASS-21, PCL-5, BES, and CRAVED scales, measured symptom severity at baseline and 4 and 12 weeks. Daily biometric data including blood glucose and ketone levels were collected.

**Results:**

Baseline measures indicated severe psychiatric symptoms, notably maximal scores for PTSD and severe binge-eating behavior. By week 12, all psychiatric symptoms resolved evidenced by quantitative reductions to 0 across all validated instruments. The patient consistently reported optimal symptom control when blood ketone levels were maintained between 3 and 5 mmol/L. Qualitative reports substantiated marked functional gains, including improved occupational engagement and social functioning.

**Conclusion:**

This report demonstrates the potential of KMT to achieve comprehensive remission in severe, treatment-resistant psychiatric comorbidities. The findings emphasize the necessity for controlled clinical trials to verify optimal therapeutic ketone ranges and establish generalizability across clinical populations experiencing complex psychiatric comorbidities.

## 1 Introduction

### 1.1 Psychiatric comorbidities: a complex challenge

Psychiatric disorders rarely occur in isolation and often present as overlapping conditions, complicating diagnosis and treatment (1). Co-occurring disorders increase symptom severity, prolong illness, impair function, and heighten treatment resistance (2–4). Multiple diagnoses often require polypharmacy, increasing the risk of adverse effects, including drug interactions and treatment non-adherence. These complications contribute to higher rates of emergency visits, hospitalizations, and overall healthcare utilization (5–8).

Certain psychiatric comorbidities occur at high rates and present distinct clinical challenges. For example, in one population study of 7,403 adults with psychiatric conditions, PTSD affected 78.5% of cases and contributed to poorer outcomes, greater symptom severity, chronicity, and increased functional impairment (9). PTSD is common in individuals with eating disorders (ED), including binge-eating disorder (BED), with prevalence of PTSD in ED populations estimated at 18%–24.6% (10). Individuals with PTSD often have comorbid ADHD, sharing overlapping symptoms such as impulsivity, emotional dysregulation, and difficulties with attention (11, 12). Moreover, those with both PTSD and ADHD are more than twice as likely to have attempted suicide than individuals with ADHD but without comorbid PTSD (13). Emerging evidence links metabolic dysfunction to symptom overlap and treatment resistance across multiple psychiatric conditions (14–16).

In PTSD, chronic stress and dysregulation of the hypothalamic-pituitary-adrenal (HPA) axis cause altered glucose metabolism, insulin resistance, and increased neuroinflammation, exacerbating symptoms such as hypervigilance, emotional dysregulation, and cognitive impairments (17). Similarly, BED is associated with metabolic dysfunctions, including impaired insulin signaling, dysregulation of appetite-related hormones like leptin and ghrelin, and alterations in dopamine pathways, which drive compulsive overeating and rewards-seeking behaviors (18–21). In ADHD, mitochondrial dysfunction has been linked to abnormalities in cellular energy metabolism and increased oxidative stress (15) and is also overrepresented among women with cardiometabolic conditions (22). Additionally, behavioral research, neuroimaging, and genetic studies have indicated a consistent neural basis for symptoms across multiple psychiatric disorders, reinforcing the biological overlap between these conditions (23). These shared pathophysiological features demonstrate the need for treatment strategies that target common underlying mechanisms rather than addressing each diagnosis separately.

### 1.2 Rethinking psychiatric treatment: the case for a transdiagnostic model

Despite high comorbidity rates, standard psychiatric treatments remain disorder-specific, often failing to address the shared underlying mechanisms that drive multiple conditions. This fragmented approach can result in ineffective treatment strategies and high symptom burden, leading to the prescription of multiple medications to target different diagnoses (24, 25). Because psychiatric disorders share overlapping symptomatology and biological mechanisms, researchers have increasingly challenged the adequacy of disorder-specific treatment approaches (25–27). Meta-analytic comparisons of PTSD treatments indicate that while certain therapies, including EMDR, show efficacy, no single approach consistently outperforms others across all patient populations in symptom reduction, remission, or treatment retention. This reinforces the need for broader, mechanism-based interventions (28, 29). Transdiagnostic models have been proposed as an alternative, targeting shared underlying mechanisms rather than treating each diagnosis separately (14, 25, 30), addressing the biological and psychological processes that contribute to multiple psychiatric disorders simultaneously (24, 25).

### 1.3 The ketogenic diet: a metabolic intervention with psychiatric applications

Given the role of metabolic dysfunction in PTSD, ADHD, and BED, a treatment targeting these mechanisms may offer a novel therapeutic approach. The ketogenic diet is one such intervention which may offer a metabolic treatment strategy for these conditions. By modulating neuroinflammation (31, 32), reducing oxidative stress (32, 33), and improving mitochondrial function (34, 35), KMT offers a metabolic treatment approach for several psychiatric conditions (36). Ketogenic diets enhance brain energy metabolism (37), increase gamma-aminobutyric acid (GABA) synthesis, and regulate glutamatergic neurotransmission by increasing the GABA/glutamate ratio, which reduces neuronal excitability (38). By addressing shared metabolic disturbances, KMT aligns with the transdiagnostic model and represents a potential avenue for improving ADHD and BED symptoms (30, 36). Early clinical reports suggest potential applications for PTSD, though direct evidence remains limited (39, 40).

Most research on KMT has focused on schizophrenia, bipolar disorder, and depression. The same transdiagnostic mechanisms targeted by KMT, including neuroinflammation, oxidative stress, and neurotransmitter imbalance, are present in PTSD, ADHD, and BED. A retrospective analysis of inpatients with severe mental illness reported substantial symptom reduction across multiple psychiatric diagnoses after KMT (41).

By stabilizing neuronal activity and improving bioenergetic efficiency, KMT may counteract treatment resistance and improve recovery in psychiatric disorders with metabolic dysfunction (42, 43). Retrospective analyses and clinical trials in psychiatric populations have reported significant symptom reductions in depression, psychosis, and anxiety after ketogenic therapy, with notable effects in treatment-resistant cases (39, 41, 44).

### 1.4 Case study introduction

Given the metabolic dysfunction underlying PTSD, ADHD, and BED and the potential for KMT to target these shared mechanisms, the following case demonstrates transdiagnostic remission across these conditions following dietary intervention. This report details the structured KMT protocol, patient’s adherence, and observed clinical outcomes.

## 2 Case presentation

### 2.1 Clinical background

A 38 years-old woman with a history of multiple psychiatric diagnoses, including PTSD, bipolar II disorder, and binge-restrict subtype eating disorder (with symptom onset at age 18), presented with severe psychological distress. She had no history of substance abuse. She reported severe depressive symptoms, including persistent low mood, cognitive impairment, and pronounced brain fog, which led to a leave of absence from her professional responsibilities. Additionally, she reported heightened anxiety, frequent panic attacks, excessive crying, disrupted sleep, and a lack of interest in and attention to her surroundings. These symptoms resulted in significant social withdrawal and an inability to fulfill professional obligations. She delayed postgraduate education and the establishment of a private practice. Overall, she reported experiencing significant physical and mental exhaustion, which limited her daily capacity. In addition to her psychiatric symptoms, she experienced lifelong cystic breasts and reported recent weight gain and poor skin health in the form of acne.

She reported a family history of bipolar I disorder and history of parental neglect, as well as physical and emotional abuse. She exhibited a longstanding history of binge-eating and restrictive behaviors, requiring multiple outpatient interventions for eating disorder treatment with limited efficacy. She self-identified as having a sugar addiction, rating it as 10/10 in severity. This was confirmed at baseline with the CRAVED questionnaire.

She engaged in trauma-focused therapy for 3 years, along with psychotherapy, which she found beneficial for emotional support and the development of internal coping resources. However, these interventions did not notably improve her symptoms. She had not trialed any psychiatric medications. She described her vision of improved mental health as the ability to experience a stable state of wellbeing and positively navigate adversity.

For 1 year prior to initiating KMT the patient followed an animal-based diet eliminating plant foods and eating only protein and fat from animal sources. This led to improvements in physical health, but suboptimal ketone levels (0.1–1.5 mmol/L) and weight gain of 20 lbs (9 kg). At baseline, her weight was 150 lbs (68 kg), and BMI was 24.9, within the healthy range. At the time of diet initiation, she was not taking any medications and had no history of medication treatment. She supplemented with sodium, potassium, and magnesium to support electrolyte balance and she adjusted this based on her needs. No adverse adaptation symptoms were reported.

### 2.2 KMT intervention strategy

The intervention included 8 h of educational content over 8 weeks, delivered by a practitioner trained in KMT implementation. Professional and peer support included two weekly calls for questions and guidance. Additional support was available through a private community platform. The program was designed to be completed in 6 months, but the patient achieved remission after 12 weeks. KMT was the only therapy she implemented during this time.

Macronutrient intake began at a 1.5:1 ratio (160 g fat, 55 g protein, 52 g net carbs) for 4 weeks, progressing to a 2:1 ratio (170 g fat, 55 g protein, 30 g net carbs) by the end of month one to optimize ketone levels. Her dietary preferences included beef, lamb, chicken, sardines, pork, eggs, dairy, and beef fat. She primarily adhered to an animal-based ketogenic diet, aligned with her food preferences. She excluded caffeinated beverages due to self-reported susceptibility to hypomanic episodes and avoided artificial and natural sweeteners.

She used the MyFitnessPal app to track macronutrient intake for approximately 1 month. During this period, focusing on caloric intake triggered eating disorder-related distress, leading to feelings of restriction and binge urges. These triggers subsided when she shifted her focus to macronutrient composition rather than calorie count. As her meals became more consistent, the need for tracking decreased but the 2:1 ratio was maintained. She also implemented intermittent fasting, restricting her eating window based on her ketone readings, to enhance ketone production. She lost 10 lbs (4.5 kg) in the first few weeks of KMT, stabilizing at 144 lbs (65 kg) by week 12.

**FIGURE 1:**
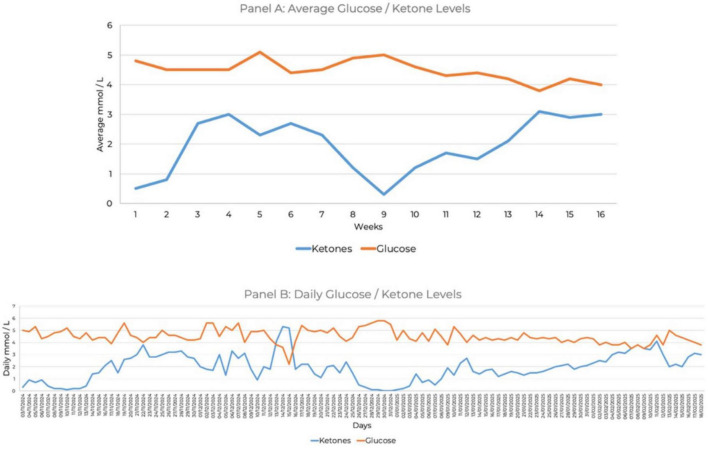
Ketone and glucose measures over 16 weeks. **(A)** Average glucose and ketone levels. **(B)** Daily glucose and ketone levels.

She maintained 100% compliance with daily ketone and glucose monitoring for 16 weeks. She tracked blood glucose and beta-hydroxybutyrate levels using the Keto-Mojo^®^ GK + blood glucose and β-ketone dual monitoring system, establishing initial ketosis at 1.4 mmol/L (Figure 1). She reported cognitive benefits, including improved mental clarity, which she described as enhanced processing speed, executive function, or attentional control, along with improvements in daily functioning when morning ketone levels ranged between 3 and 5 mmol/L. Ketone levels between 1 and 2 mmol/L provided some cognitive and functional benefits, but she noted a marked improvement at or above 3 mmol/L. During week 9 of the intervention, which coincided with the holiday season, her ketone levels decreased due to increased carbohydrate intake (Figure 1).

## 3 Evaluation of intervention outcomes

### 3.1 Quantitative analysis

Psychiatric assessments for depression (PHQ-9), anxiety (GAD-7), stress (DASS-21), food addiction behaviors (CRAVED), binge-eating symptoms (BES), and PTSD symptoms (PCL-5) were administered at baseline and during KMT treatment with specific time-points detailed below.

The CRAVED is an ICD-10-based food-behavior questionnaire that screens for food addiction symptoms (45, 46). The CRAVED was measured at baseline. The Binge Eating Scale (BES) is a widely used, reliable and validated screening and assessment tool that evaluates the severity and frequency of binge-eating episodes (47). The PTSD Checklist for DSM-5 (PCL-5) assesses PTSD symptom severity (48). These measures were administered at baseline and at 12 weeks.

The Patient Health Questionnaire-9 (PHQ-9) is a reliable and validated diagnostic tool for assessing the severity and presence of depressive symptoms (49). The Generalized Anxiety Scale-7 (GAD-7) is widely used as a screening tool for anxiety severity (50), as well as for other anxiety disorders such as panic, social anxiety disorder, and post-traumatic stress disorder (51). The Depression, Anxiety, and Stress Scale-21 (DASS-21) measures the severity of symptoms related to depression, anxiety and stress. It is often used to assess the level of treatment response (52). These assessments were conducted at baseline and 4 and 12 weeks to track changes over time.

At baseline, the PHQ-9 score was 27, indicating severe depression. At 4 weeks, the PHQ-9 score decreased to seven, indicating mild depression. By week 12, the PHQ-9 score was 0, indicating full remission of depressive symptoms. The GAD-7 anxiety score at baseline was 16, indicating severe anxiety. At week 4, the score reduced to mild anxiety with a score of six, and by week 12, full remission of anxiety was achieved with a GAD-7 score of zero. The baseline DASS-21 showed a depression score of 21 (severe), an anxiety score of seven (mild), and a stress score of 12 (mild), with a total DASS score of 80. At week 4, depression, anxiety, and stress had all reduced to normal levels with depression scores of four, anxiety scores of two and stress scores of one, for a total of 14. By week 12, depression, anxiety, and stress scores had all dropped to zero, indicating full remission.

**FIGURE 2:**
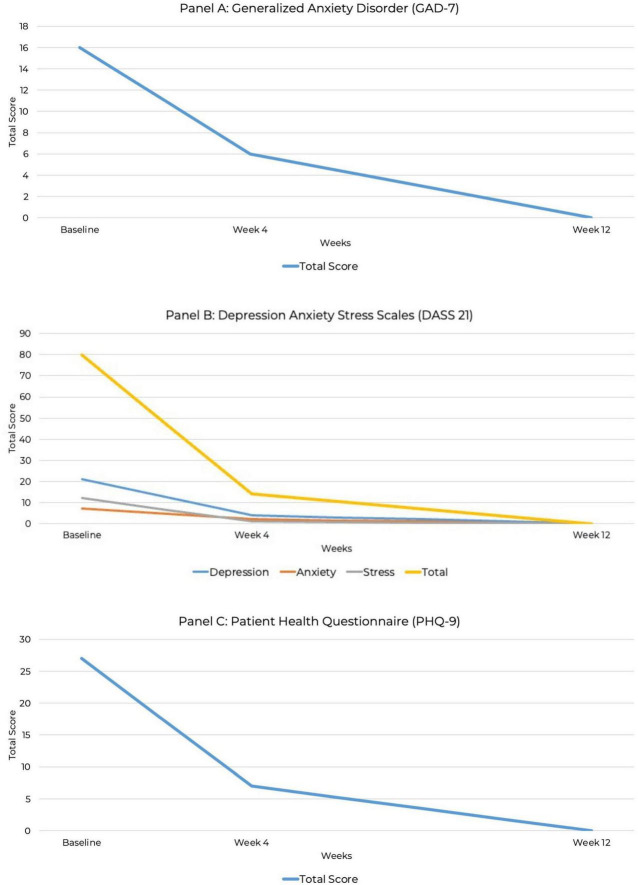
Line graph depicting the reduction in GAD-7, DASS-21, and PHQ-9 total scores over 12 weeks. **(A)** Generalized anxiety disorder (GAD-7). **(B)** Depression Anxiety Stress Scale (DASS-21). **(C)** Patient Health Questionnaire (PHQ-9).

**FIGURE 3:**
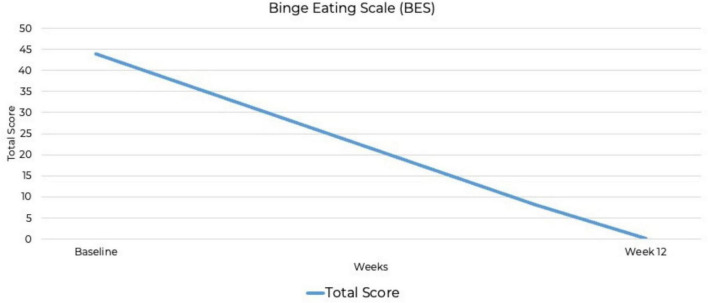
Improvement in binge-eating symptoms over 12 weeks Binge Eating Scale (BES).

**FIGURE 4:**
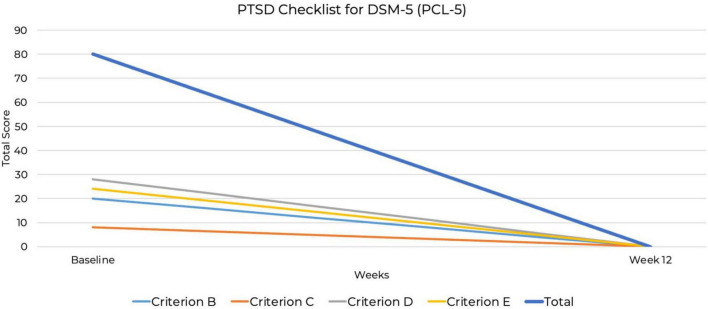
Decrease in post-traumatic stress disorder (PTSD) symptoms over 12 weeks PTSD Checklist for DSM-5 (PCL-5).

At baseline, the PCL-5 score was 80, the maximum possible score on the measure. Although the PCL-5 does not include standardized severity ranges, this score suggests severe PTSD symptomatology. By week 12, the PCL-5 score had dropped to zero, indicating full remission. This represents the most substantial improvement, from the highest possible PCL-5 score to zero, indicating complete symptom remission. A baseline BES score of 44 out of a possible 46 indicated severe binge-eating behavior (53, 54), along with a six out of six CRAVED score, indicating a potential substance use disorder. By week 12, the BES score had dropped to zero out of 46, indicating no binge-eating behavior and full remission. By week 12, scores on all psychiatric assessments had decreased to zero, indicating full remission of symptoms (Figures 2–4). At the point of writing this case study, she continues to adhere to KMT to sustain her therapeutic progress.

### 3.2 Qualitative analysis

The patient experienced marked improvements in mental health, with increased emotional resilience and greater stability in her psychological wellbeing:

*“All my mental health symptoms have improved significantly. While I still have difficult moments, I feel so much better equipped to deal with them. I have a sense of well-being now which ensures I can function well each day regardless of my mood.”*

She noted improvements in physical health and energy levels, making prioritizing her health more attainable:

*“I am feeling much more alert and focused, and my passion for my work has returned. My physical health feels good, I would like to start exercising and improve my sleep cycle. This feels more possible as I notice my energy improving.”*

She also reported restored motivation and an increase in confidence and self-efficacy:

*“I have returned to work (agency work) and started up my private practice. This was a dream for the last year that I could not realize until now. I can focus much better on my work and am receiving positive feedback which feels great.”*

These findings align with previous studies reporting that participants experienced improvements in mood, energy levels, hunger regulation, cravings, and overall psychological wellbeing (39, 55–60).

Over the holidays, her ketones decreased due to consuming more carbohydrates. She noted that “*It takes me 3 days to get back into ketosis”* while adhering to her macronutrient ratios. This is in keeping with the literature, which indicates that it can take up to 84 h to achieve ketosis with a therapeutic ketogenic diet (61). During this time, she experienced a deterioration in her mental health and an increase in negative symptoms:

*“Once I’m in ketosis I feel great, when I fall out due to sugar etc., my symptoms return rapidly. When I’m out of ketosis the impact is drastic.”*

This is consistent with previous research, where participants reported that increasing carbohydrate intake and deviating from the dietary plan increased their hunger and cravings. Additionally, these changes negatively impacted their mood and physical health (55). The patient stated:

*“I realize I need to treat ketosis with the same seriousness as medication. The metabolic shift back to glucose is profound and devastating, as I imagine it could be if someone stopped potent medication abruptly.”*

She also struggled with her “*apparent addiction to sugar”* while implementing the diet. However, KMT appeared to provide her with marked relief:

“*Sugar has been an issue throughout. Since KMT, I have seen rapid improvements within days to weeks. The only inhibitor is my apparent addiction to sugar.”*

Her reported experience aligns with recent studies in which participants experienced complete resolution of their sugar addiction and binge behaviors with a ketogenic diet (55, 56, 62, 63).

## 4 Discussion

### 4.1 Summary of findings

This case report describes the remission of PTSD, ADHD, and BED symptoms in a patient following a structured KMT intervention. Findings are consistent with previous reports. Quantitative assessments using the PHQ-9, GAD-7, DASS-21, PCL-5, BES, and CRAVED scales indicated full symptom resolution within a 12 weeks period. The individual consistently monitored blood ketone and glucose levels and reported optimal mental health functioning specifically when beta-hydroxybutyrate (BHB) concentrations exceeded 3 mmol/L. Throughout the majority of the intervention, she consistently maintained therapeutic levels of ketosis in the morning (1.5–3 mmol/L) with ketone concentrations increasing over the course of the day. reports further highlighted the patient’s perception of symptom recurrence correlating directly with periods of reduced ketosis, with sugar intake being the main driver of lower ketones. These observations suggest a potential dose-response relationship between higher ketone levels and psychiatric symptom management within this case, warranting further investigation in clinical trials.

### 4.2 Comparison with existing literature

A recent case series of three patients with complex psychiatric presentations reported significant improvements in depressive and anxiety symptoms following ketogenic dietary interventions, as measured by the PHQ-9 and GAD-7, respectively (39). Although two participants in this series had comorbid PTSD diagnoses, PTSD-specific symptom measures such as the PCL-5 were not utilized. The current case report explicitly assessed PTSD symptom severity using the PCL-5 and documented full remission, from the highest possible severity score to 0 within a 12-week period, in a patient presenting with additional psychiatric comorbidities. This observation expands upon preliminary evidence supporting the use of KMT for PTSD for complex psychiatric presentations, although the presence of additional comorbidities limits interpretation specific to PTSD alone.

No formally published human case reports have specifically documented outcomes related to ADHD symptoms using KMT. While the current case did not administer validated ADHD symptom measures, the patient had a documented clinical history of ADHD diagnosis with related functional impairments and qualitatively reported improvements in attention, focus, and emotional regulation, which overlap clinically with recognized ADHD symptom domains (64). Although these subjective improvements suggest potential ADHD-related benefits, the absence of formal ADHD-specific assessments limits interpretation regarding symptom severity or remission. Future controlled investigations should explicitly evaluate ADHD symptoms using validated instruments to determine the relevance of KMT for this patient population.

One previous case series of three participants documented reductions in binge-eating symptoms as measured by the BES, food addiction symptoms as measured by the Yale Food Addiction Scale, and depressive symptoms assessed using the PHQ-9 following a ketogenic dietary intervention explicitly comprising 60% fat, 30% protein, and 10% carbohydrates maintained consistently over 6–7 months (62), with participants reporting therapeutic blood ketone levels of 0.5–5.0 mmol/L. In a pilot trial, four out of five participants diagnosed with binge-eating and/or food addiction behaviors measured by the BES and Yale Food Addiction Scale reported remission of symptoms following 7 weeks on a very low-calorie ketogenic diet, which included an energy intake of 1,000 kcal per day and carbohydrate consumption of < 25 g per day (63). A review on KMT use in binge eating and ultra-processed food addiction suggests that it could be considered a treatment approach due to the ability to abstain from addictive-like foods (65).

The current case report documented remission of these symptoms within a notably shorter 12 weeks timeframe using a ketogenic diet initially comprising approximately 77% fat, 12% protein, and 11% carbohydrates, progressing to approximately 82% fat, 12% protein, and 6% carbohydrates within the first month. Moreover, symptom remission in this case was reported with a narrower and consistently higher therapeutic ketone range of 3.0–5.0 mmol/L. This report also included assessments of PTSD and generalized anxiety disorder using validated measures, expanding the scope of psychiatric symptoms formally evaluated beyond those reported previously.

Despite clear documentation of psychiatric symptom remission in this case, the inherent methodological limitations of single case reports require explicit acknowledgment. This case alone cannot establish definitive therapeutic ketone thresholds nor reliably predict clinical outcomes across broader patient populations. Controlled clinical trials are necessary to replicate these findings and confirm therapeutic efficacy. Additionally, future research should incorporate qualitative methods to capture detailed patient experiences, adherence behaviors, and barriers to sustained ketosis. This integrative approach will facilitate the development of clinically meaningful, evidence-informed KMT treatment protocols.

## 5 Conclusion

This case report documents significant remission of psychiatric symptoms, formally assessed through quantitative measures in PTSD and BED following KMT. Additionally, the patient qualitatively reported meaningful improvements in attention, focus, emotional regulation, and occupational and social functioning, consistent with recovery from a formally diagnosed ADHD condition. These combined quantitative and qualitative outcomes strongly support KMT’s potential as a targeted intervention addressing shared biological mechanisms underlying complex psychiatric presentations. Future research should further investigate these findings across diverse psychiatric populations.

## Data Availability

The original contributions presented in this study are included in this article/supplementary material, further inquiries can be directed to the corresponding author.

## References

[1] Garakani A. Commentary: Diagnostic challenges. *J Psychiatr Pract.* (2022) 28:270. 10.1097/PRA.0000000000000630 35511106

[2] van Kranenburg G Diekman W Mulder W Pijnenborg G van den Brink R Mulder C. Histories of social functioning and mental healthcare in severely dysfunctional dual-diagnosis psychiatric patients. *Int J Ment Health Addict.* (2020) 18:904–16. 10.1007/s11469-018-9992-7

[3] Ecker A Cully J Cucciare M Hundt N. Patient and provider perspectives on treating substance use disorder and co-occurring anxiety and posttraumatic stress disorders in the veterans affairs healthcare system. *J Veterans Stud.* (2023) 9:171–80. 10.21061/jvs.v9i1.403

[4] Howes O Thase M Pillinger T. Treatment resistance in psychiatry: State of the art and new directions. *Mol Psychiatry.* (2022) 27:58–72. 10.1038/s41380-021-01200-3 34257409 PMC8960394

[5] Doumat G Daher D Itani M Abdouni L El Asmar K Assaf G. The effect of polypharmacy on healthcare services utilization in older adults with comorbidities: A retrospective cohort study. *BMC Prim Care.* (2023) 24:120. 10.1186/s12875-023-02070-0 37237338 PMC10214698

[6] Benz M Epstein-Lubow G Weinstock L Gaudiano B. Polypharmacy Among patients with major depressive disorder and co-occurring substance use disorders in a psychiatric hospital setting: Prevalence and risk factors. *J Clin Psychopharmacol.* (2023) 43:273. 10.1097/JCP.0000000000001681 37039705 PMC11005319

[7] Pati S Mahapatra P Dwivedi R Athe R Sahoo K Samal M Multimorbidity and its outcomes among patients attending psychiatric care settings: An observational study from Odisha, India. *Front Public Health.* (2021) 8:616480. 10.3389/fpubh.2020.616480 33968863 PMC8096979

[8] van Schijndel M Jansen L Busschbach J van Waarde J Wierdsma A Tiemeier H. Hospital healthcare utilizers with medical and psychiatric claims in the Netherlands: A nationwide study. *BMC Health Serv Res.* (2022) 22:480. 10.1186/s12913-022-07798-6 35410295 PMC9004012

[9] Qassem T Aly-ElGabry D Alzarouni A Abdel-Aziz K Arnone D. Psychiatric co-morbidities in post-traumatic stress disorder: Detailed findings from the adult psychiatric morbidity survey in the English population. *Psychiatr Q.* (2021) 92:321–30. 10.1007/s11126-020-09797-4 32705407 PMC7904722

[10] Ferrell E Russin S Flint D. Prevalence estimates of comorbid eating disorders and posttraumatic stress disorder: A quantitative synthesis. *J Aggress Maltreatment Trauma.* (2022) 31:264–82. 10.1080/10926771.2020.1832168

[11] Punski-Hoogervorst J Engel-Yeger B Avital A. Attention deficits as a key player in the symptomatology of posttraumatic stress disorder: A review. *J Neurosci Res.* (2023) 101:1068–85. 10.1002/jnr.25177 36807926

[12] Piszczor J Shoemaker B Jones M Soble JR. B - 126 posttraumatic stress disorder symptoms as a predictor of invalid attention-deficit/hyperactivity disorder and general psychiatric symptom reporting among adult neuropsychology referrals. *Arch Clin Neuropsychol.* (2024) 39:1229. 10.1093/arclin/acae067.287

[13] Ziegler G Groß S Boreatti A Heine M McNeill R Kranz T Suicidal behavior in ADHD: The role of comorbidity, psychosocial adversity, personality and genetic factors. *Discov Ment Health.* (2024) 4:51. 10.1007/s44192-024-00103-3 39499453 PMC11538115

[14] Anderson J Ozan E Chouinard V Grant G MacDonald A Thakkar L The ketogenic diet as a transdiagnostic treatment for neuropsychiatric disorders: Mechanisms and clinical outcomes. *Curr Treat Options Psychiatry.* (2024) 12:1. 10.1007/s40501-024-00339-4

[15] Öğütlü H Kaşak M Tabur S. Mitochondrial dysfunction in attention deficit hyperactivity disorder. *Eurasian J Med.* (2022) 54:S187–95. 10.5152/eurasianjmed.2022.22187 36655466 PMC11163340

[16] Monda A La Torre M Messina A Di Maio G Monda V Moscatelli F Exploring the ketogenic diet’s potential in reducing neuroinflammation and modulating immune responses. *Front Immunol.* (2024) 15:1425816. 10.3389/fimmu.2024.1425816 39188713 PMC11345202

[17] Song Y Baranova A Cao H Yue W Zhang F. Causal associations between posttraumatic stress disorder and type 2 diabetes. *Diabetol Metab Syndr.* (2025) 17:63. 10.1186/s13098-025-01630-x 39972391 PMC11837430

[18] Berner L Brown T Lavender J Lopez E Wierenga C Kaye W. Neuroendocrinology of reward in anorexia nervosa and bulimia nervosa: Beyond leptin and ghrelin. *Mol Cell Endocrinol.* (2019) 497:110320. 10.1016/j.mce.2018.10.018 30395874 PMC6497565

[19] Baenas I Miranda-Olivos R Solé-Morata N Jiménez-Murcia S Fernández-Aranda F. Neuroendocrinological factors in binge eating disorder: A narrative review. *Psychoneuroendocrinology.* (2023) 150:106030. 10.1016/j.psyneuen.2023.106030 36709632

[20] Yu Y Miller R Groth SWA. literature review of dopamine in binge eating. *J Eat Disord.* (2022) 10:11. 10.1186/s40337-022-00531-y 35090565 PMC8796589

[21] Rania M Caroleo M Carbone E Ricchio M Pelle M Zaffina I Reactive hypoglycemia in binge eating disorder, food addiction, and the comorbid phenotype: Unravelling the metabolic drive to disordered eating behaviours. *J Eat Disord.* (2023) 11:162. 10.1186/s40337-023-00891-z 37726785 PMC10507855

[22] Smari U Valdimarsdottir U Aspelund T Hauksdottir A Thordardottir E Hartman C Psychiatric comorbidities in women with cardiometabolic conditions with and without ADHD: A population-based study. *BMC Med.* (2023) 21:450. 10.1186/s12916-023-03160-7 37981673 PMC10659052

[23] Xie C Xiang S Shen C Peng X Kang J Li Y A shared neural basis underlying psychiatric comorbidity. *Nat Med.* (2023) 29:1232–42. 10.1038/s41591-023-02317-4 37095248 PMC10202801

[24] Nolen-Hoeksema S Watkins ERA. heuristic for developing transdiagnostic models of psychopathology: Explaining multifinality and divergent trajectories. *Perspect Psychol Sci.* (2011) 6:589–609. 10.1177/1745691611419672 26168379

[25] Kotov R Krueger R Watson D Achenbach T Althoff R Bagby R The hierarchical taxonomy of psychopathology (HiTOP): A dimensional alternative to traditional nosologies. *J Abnorm Psychol.* (2017) 126:454–77. 10.1037/abn0000258 28333488

[26] Arribas M Oliver D Patel R Kornblum D Shetty H Damiani S A transdiagnostic prodrome for severe mental disorders: An electronic health record study. *Mol Psychiatry.* (2024) 29:3305–15. 10.1038/s41380-024-02533-5 38710907 PMC11540905

[27] Antuña-Camblor C Peris-Baquero Ó Juarros-Basterretxea J Cano-Vindel A Rodríguez-Díaz FJ. Transdiagnostic risk factors of emotional disorders in adults: A systematic review. *An Psicol Ann Psychol.* (2024) 40:199–218. 10.6018/analesps.561051

[28] Yunitri N Chu H Kang X Wiratama B Lee T Chang L Comparative effectiveness of psychotherapies in adults with posttraumatic stress disorder: A network meta-analysis of randomised controlled trials. *Psychol Med.* (2023) 53:6376–88. 10.1017/S0033291722003737 36628572

[29] Wright S Karyotaki E Cuijpers P Bisson J Papola D Witteveen A EMDR v. other psychological therapies for PTSD: A systematic review and individual participant data meta-analysis. *Psychol Med.* (2024) 54:1580–8. 10.1017/S0033291723003446 38173121

[30] Hoy N Lynch S Waszczuk M Reppermund S Mewton L. Transdiagnostic biomarkers of mental illness across the lifespan: A systematic review examining the genetic and neural correlates of latent transdiagnostic dimensions of psychopathology in the general population. *Neurosci Biobehav Rev.* (2023) 155:105431. 10.1016/j.neubiorev.2023.105431 37898444

[31] Fu S Li S Wang J Li Y Xie S Xue W BHBA suppresses LPS-induced inflammation in BV-2 cells by inhibiting NF-κB activation. *Mediat Inflamm.* (2014) 2014:983401. 10.1155/2014/983401 24803746 PMC3997897

[32] Morris G Puri B Maes M Olive L Berk M Carvalho A. The role of microglia in neuroprogressive disorders: Mechanisms and possible neurotherapeutic effects of induced ketosis. *Prog Neuropsychopharmacol Biol Psychiatry.* (2020) 99:109858. 10.1016/j.pnpbp.2020.109858 31923453

[33] Haces M Hernández-Fonseca K Medina-Campos O Montiel T Pedraza-Chaverri J Massieu L. Antioxidant capacity contributes to protection of ketone bodies against oxidative damage induced during hypoglycemic conditions. *Exp Neurol.* (2008) 211:85–96. 10.1016/j.expneurol.2007.12.029 18339375

[34] Vidali S Aminzadeh S Lambert B Rutherford T Sperl W Kofler B Mitochondria: The ketogenic diet–a metabolism-based therapy. *Int J Biochem Cell Biol.* (2015) 63:55–9. 10.1016/j.biocel.2015.01.022 25666556

[35] Miller V LaFountain R Barnhart E Sapper T Short J Arnold W A ketogenic diet combined with exercise alters mitochondrial function in human skeletal muscle while improving metabolic health. *Am J Physiol Endocrinol Metab.* (2020) 319:E995–1007. 10.1152/ajpendo.00305.2020 32985255

[36] Mentzelou M Dakanalis A Vasios G Gialeli M Papadopoulou S Giaginis C. The relationship of ketogenic diet with neurodegenerative and psychiatric diseases: A scoping review from basic research to clinical practice. *Nutrients.* (2023) 15:2270. 10.3390/nu15102270 37242153 PMC10220548

[37] Jensen N Wodschow H Nilsson M Rungby J. Effects of ketone bodies on brain metabolism and function in neurodegenerative diseases. *Int J Mol Sci.* (2020) 21:8767. 10.3390/ijms21228767 33233502 PMC7699472

[38] Qiao Y Li L Hu S Yang Y Ma Z Huang L Ketogenic diet-produced β-hydroxybutyric acid accumulates brain GABA and increases GABA/glutamate ratio to inhibit epilepsy. *Cell Discov.* (2024) 10:1–20. 10.1038/s41421-023-00636-x 38346975 PMC10861483

[39] Calabrese L Frase R Ghaloo M. Complete remission of depression and anxiety using a ketogenic diet: Case series. *Front Nutr.* (2024) 11:1396685. 10.3389/fnut.2024.1396685 38887496 PMC11182043

[40] Edwards M Furuholmen-Jenssen T Søegaard E Thapa S Andersen J. Exploring diet-induced ketosis with exogenous ketone supplementation as a potential intervention in post-traumatic stress disorder: A feasibility study. *Front Nutr.* (2024) 11:1406366. 10.3389/fnut.2024.1406366 39588043 PMC11586679

[41] Danan A Westman E Saslow L Ede G. The ketogenic diet for refractory mental illness: A retrospective analysis of 31 inpatients. *Front Psychiatry.* (2022) 13:951376. 10.3389/fpsyt.2022.951376 35873236 PMC9299263

[42] Freyberg Z Andreazza A McClung C Phillips M. Linking mitochondrial dysfunction, neurotransmitter, and neural network abnormalities and mania: Elucidating neurobiological mechanisms of the therapeutic effect of the ketogenic diet in bipolar disorder. *Biol Psychiatry Cogn Neurosci Neuroimaging.* (2024) 10:267–77. 10.1016/j.bpsc.2024.07.011 39053576 PMC11754533

[43] Kovács Z Brunner B Ari C. Beneficial effects of exogenous ketogenic supplements on aging processes and age-related neurodegenerative diseases. *Nutrients.* (2021) 13:2197. 10.3390/nu13072197 34206738 PMC8308443

[44] Sethi S Wakeham D Ketter T Hooshmand F Bjornstad J Richards B Ketogenic diet intervention on metabolic and psychiatric health in bipolar and schizophrenia: A pilot trial. *Psychiatry Res.* (2024) 335:115866. 10.1016/j.psychres.2024.115866 38547601

[45] Unwin J Delon C Giæver H Kennedy C Painschab M Sandin F Low carbohydrate and psychoeducational programs show promise for the treatment of ultra-processed food addiction. *Front Psychiatry.* (2022) 13:1005523. 10.3389/fpsyt.2022.1005523 36245868 PMC9554504

[46] Unwin J Delon C Giaever H Kennedy C Painschab M Sandin F Low carbohydrate and psychoeducational programs show promise for the treatment of ultra-processed food addiction: 12-month follow-up. *Front Psychiatry.* (2025) 16:1556988. 10.3389/fpsyt.2025.1556988 40357512 PMC12067479

[47] Gormally J Black S Daston S Rardin D. The assessment of binge eating severity among obese persons. *Addict Behav.* (1982) 7:47–55. 10.1016/0306-4603(82)90024-7 7080884

[48] Ashbaugh A Houle-Johnson S Herbert C El-Hage W Brunet A. Psychometric validation of the english and french versions of the posttraumatic stress disorder checklist for DSM-5 (PCL-5). *PLoS One.* (2016) 11:e0161645. 10.1371/journal.pone.0161645 27723815 PMC5056703

[49] Kroenke K Spitzer R Williams J. The PHQ-9: Validity of a brief depression severity measure. *J Gen Intern Med.* (2001) 16:606–13. 10.1046/j.1525-1497.2001.016009606.x 11556941 PMC1495268

[50] Spitzer R Kroenke K Williams J Löwe B. A brief measure for assessing generalized anxiety disorder: The GAD-7. *Arch Intern Med.* (2006) 166:1092–7. 10.1001/archinte.166.10.1092 16717171

[51] Kroenke K Spitzer R Williams J Monahan P Löwe B. Anxiety disorders in primary care: Prevalence, impairment, comorbidity, and detection. *Ann Intern Med.* (2007) 146:317–25. 10.7326/0003-4819-146-5-200703060-00004 17339617

[52] Lovibond P Lovibond S. The structure of negative emotional states: Comparison of the depression anxiety stress scales (DASS) with the Beck depression and anxiety inventories. *Behav Res Ther.* (1995) 33:335–43. 10.1016/0005-7967(94)00075-u 7726811

[53] Marcus M Wing R Lamparski D. Binge eating and dietary restraint in obese patients. *Addict Behav.* (1985) 10:163–8. 10.1016/0306-4603(85)90022-X 3859990

[54] Duarte C Pinto-Gouveia J Ferreira C. Expanding binge eating assessment: Validity and screening value of the Binge Eating Scale in women from the general population. *Eat Behav.* (2015) 18:41–7. 10.1016/j.eatbeh.2015.03.007 25880043

[55] Bellamy E Hadjiefthyvoulou F Walsh J Brown J Turner J. Understanding the experiences of ketogenic metabolic therapy for people living with varying levels of depressive symptoms: A thematic analysis. *Front Nutr.* (2024) 11:1397546. 10.3389/fnut.2024.1397546 38903620 PMC11188922

[56] Campbell I Kamenska I Bellamy E. *Bipolar disorder and ketogenic diet a survey of the lived experience of over 100 patients.* Edinburgh: University of Edinburgh (2024). 10.31234/osf.io/6kcdx

[57] Campbell I Campbell H. Ketosis and bipolar disorder: Controlled analytic study of online reports. *BJPsych Open.* (2019) 5:e58. 10.1192/bjo.2019.49 31530294 PMC6620566

[58] Laurent N. Retrospective case study: Ketogenic metabolic therapy in the effective management of treatment-resistant depressive symptoms in bipolar disorder. *Front Nutr.* (2024) 11:1394679. 10.3389/fnut.2024.1394679 39188977 PMC11346312

[59] Laurent N Bellamy E Tague K Hristova D Houston A. Ketogenic metabolic therapy for schizoaffective disorder: A retrospective case series of psychotic symptom remission and mood recovery. *Front Nutr.* (2025) 12:1506304. 10.3389/fnut.2025.1506304 39990610 PMC11844221

[60] Laurent N Bellamy E Hristova D Houston A. Ketogenic metabolic therapy in the remission of chronic major depressive disorder: A retrospective case study. *Front Nutr.* (2025) 12:1549782. 10.3389/fnut.2025.1549782 40083888 PMC11903285

[61] Wirrell E Darwish H Williams-Dyjur C Blackman M Lange V. Is a fast necessary when initiating the ketogenic diet? *J Child Neurol.* (2002) 17:179–82. 10.1177/088307380201700305 12026232

[62] Carmen M Safer D Saslow L Kalayjian T Mason A Westman E Treating binge eating and food addiction symptoms with low-carbohydrate Ketogenic diets: A case series. *J Eat Disord.* (2020) 8:2. 10.1186/s40337-020-0278-7 32010444 PMC6988301

[63] Rostanzo E Marchetti M Casini I Aloisi A. Very-low-calorie ketogenic diet: A potential treatment for binge eating and food addiction symptoms in women. A pilot study. *Int J Environ Res Public Health.* (2021) 18:12802. 10.3390/ijerph182312802 34886528 PMC8657275

[64] American Psychiatric Association. *Diagnostic and statistical manual of mental disorders: DSM-5.* 5th ed. Washington, DC: American Psychiatric Publishing (2013).

[65] Sethi S Sinha A Gearhardt A. Low carbohydrate ketogenic therapy as a metabolic treatment for binge eating and ultraprocessed food addiction. *Curr Opin Endocrinol Diabetes Obes.* (2020) 27:275. 10.1097/MED.0000000000000571 32773576

